# Demographics and economic burden of un-owned cats and dogs in the UK: results of a 2010 census

**DOI:** 10.1186/1746-6148-8-163

**Published:** 2012-09-13

**Authors:** Jenny Stavisky, Marnie L Brennan, Martin Downes, Rachel Dean

**Affiliations:** 1 Centre for Evidence-based Veterinary Medicine, School of Veterinary Medicine and Science, The University of Nottingham, Sutton Bonington Campus, Loughborough LE12 5RD, UK

## Abstract

**Background:**

The population of dogs and cats passing through rescue shelters may be subject to compromised welfare and increased susceptibility to disease. Little information exists to describe this population, its dynamics and associated management practices. The aim of this study was to carry out a census of un-owned cats and dogs in the UK in 2010, and to document the origins, destinations, husbandry and costs associated with the care of these animals.

**Results:**

A sampling frame was constructed by searching the databases of publicly registered charities for England, Scotland and Wales, registers of breed rescues, and by internet searches of animal welfare websites. Overall, 2,352 contacts for 1,380 organisations were identified. All were sent a postal questionnaire asking for data on the number of dogs and cats housed, their origins and eventual outcomes, and details of husbandry between January 1^st^ and December 31^st^ 2010. For those which were registered charities (595), financial records were also obtained.

A response rate of 38.8% was obtained. Overall, in 2010, 89,571 dogs and 156,826 cats entered the care of the participating organisations. Approximately half of these animals were relinquished by their owners. Other origins included being found as strays or confiscated for welfare purposes. Seventy-five per cent of dogs and 77.1% of cats were rehomed. The next most common outcome was euthanasia, accounting for 10.4% of dogs and 13.2% cats. For dogs and cats, 44.3% and 62% of participants respectively reported having a waiting list, which frequently exceeded the actual capacity of the facility. Over 19,000 people were involved in the care of these animals, on a paid or voluntary basis. Financial records were available for 519/595 (87.2%) of the registered charities, and their total expenditure in 2010 was £340 million.

**Conclusions:**

This study showed that a large number of animals become un-owned each year, which could have considerable implications for their welfare. Despite the resources expended, demand still exceeds capacity for many organisations, and a substantial number of both cats and dogs are euthanased, suggesting that further understanding of how and why these animals become un-owned is essential in order to target interventions.

## Background

It has been estimated that there are approximately 10 million pet dogs and the same number of pet cats in the UK [1], with around one third of these having been acquired from a shelter or welfare organisation [2]. It is therefore evident that there are large numbers of animals passing through rescue organisations each year. However, there are few sources of data regarding the make-up of this population and its inter-relationship with the owned population. One such source is the data from local authorities, which have a statutory requirement to collect stray dogs in the UK. Dogs Trust collates these figures and produces an annual report [3], which showed that an estimated 121,693 stray dogs were collected by local authorities in 2010. A quarter of these dogs were transferred to private shelter organisations. Additionally, a small number of shelter organisations publish their own figures, although the majority do not. A recent survey estimated the number of dogs and cats entering UK shelters in 2009 to be 129,743 and 131,070 respectively [4], showing the un-owned pet problem is one of considerable scale.

Worldwide, attempts have been made to characterise populations of un-owned cats and dogs. The Shelter Statistics Study in the US was an attempt to collect more information about the un-owned population, and encountered substantial logistical problems in attempting to co-ordinate data collection within and between these busy environments [5]. The Asilomar Accords in the United States represented an effort to co-ordinate the collection and sharing of shelter data [6], but they have only been partially successful, and remain controversial. There remains what has been described as a “statistical black hole” [7] regarding baseline population data for this large and vulnerable population of animals. In Sweden, a survey of cat shelters [8] revealed an estimated intake of 5,600 cats per year, chiefly from the stray population. Similarly, a study of 15,206 cats admitted to a shelter in Australia found that 81.6% were strays or ‘semi-owned’ animals [9]. Uncontrolled reproduction appears to be a likely contributing factor, at least for cats, with one report in the UK finding that 19% of pet cats had had at least one unplanned litter [1]. Relinquishment by pet owners has also been reported to be a significant contributor; in one US study, 4.4% and 3.8% of dog- and cat-owning households respectively had relinquished a pet to a shelter in the previous year [10]. A small number of studies have investigated the reasons why animals are relinquished to such organisations. There appears to be correlation with the owner’s lifestyle, education and expectations of owning pet, as well as true or perceived behavioural problems in the animals themselves [11-16]. Animals in a shelter are subject to numerous outcomes, including reclaim by the original owner, adoption by a new owner, release (if feral), death or living on a long-term basis in the shelter [4]. Therefore, there is a complex inter-relationship between the owned and un-owned population.

It has been suggested that un-owned cats and dogs, living as strays or in shelters, are at an increased risk of impaired physical and psychological welfare [17,18]. Added to this, they are likely to be at an increased risk of some infectious diseases when compared to the owned pet population [19-21]. Diseases which emerge in the un-owned population have potential to affect the owned population, when a formerly un-owned animal is adopted, or via direct or indirect contact. This has been seen recently in outbreaks of virulent systemic feline calicivirus in cats [22] and *Streptococcus equi* pneumonia in dogs [23].

It has been suggested that the recent economic downturn may be increasing the demands placed on shelters by increasing relinquishment and reducing rehoming of animals [24]. A clearer understanding of the dynamics of this population, and its relationship with the owned population would enable better targeting of resources to maximise the potential improvement in animal welfare.

The aims of this study were: to create a complete sampling frame of cat and dog shelter organisations within the UK; to carry out a census of the population of un-owned animals administered by these organisations; and to document the origins, destinations, husbandry and costs associated with the care of these animals in 2010.

## Methods

### Sampling frame

A combination of methods was utilised to construct a sampling frame of organisations housing and/or rehoming cats or dogs. An initial search was made using national registers of charitable organisations. For English and Welsh organisations, the Charity Commission website was searched [25]. For Scotland, the online records of the Office of the Scottish Charity Regulator (OSCR) were searched [26]. For Northern Ireland, no comparable register was available at the time of the study. For both the Charity Commission and the OSCR, the websites were queried for organisations whose records contained one or more of the terms “cat” or ”dog” or “canine” or “feline” or “animal”. For the OSCR, the additional option ‘purposes-advancement of animal welfare’ was ticked to avoid the return of irrelevant words such as ‘cathedral’.

All organisations whose scope included housing or re-homing (dogs or cats) or trap-neuter-return (cats) within the UK were admitted to the sampling frame. If it was not possible to clearly establish the scope of the organisation from the register, a check of the organisation’s website was performed, where available. Several of the large, national organisations had multiple branches, and these were approached centrally to obtain both complete lists of contacts, and overall organisational data, in addition to branch data. Where it appeared that a single organisation had multiple contacts (e.g. similar names), all were contacted. Subsequently, any replies containing duplicated data were excluded, and all non-duplicates were treated as independent sampling points. Lists of breed-specific rescues were also obtained from both the Kennel Club and the Governing Council of the Cat Fancy.

Further organisations were identified using a combination of animal welfare websites. These were initially identified via Google, using search terms including “dog”, “cat”, “animal”, “rescue” and “shelter” in various permutations. The sampling frame was expanded during the study, using a form of snowball sampling [27], as respondents provided the names of other web directories, organisations and individuals known to them.

### Questionnaire design and distribution

A questionnaire was designed using an automated reading software programme (Cardiff TeleForm, Autonomy Cardiff). The questionnaire consisted of open and closed questions with space for respondents to make further comments as necessary. Data collected included: the numbers of animals, husbandry information, sources and eventual fate of dogs and cats cared for, and numbers of staff. Participants were asked to give information about the animals under their care from the beginning of January 2010 to the end of December 2010. A copy of the questionnaire is available at http://www.nottingham.ac.uk/cevm/documents/sheltermedicine/pupsquestionnaire.pdf.

All organisations identified were contacted, and asked to fill in the questionnaire. Postal addresses were available for most of the sampling frame. If there was no postal address, the telephone number was used if available to contact the organisation to obtain a postal address. Telephone contact was attempted on at least three separate occasions at different times of the day. Organisations with no postal or telephone contact details were contacted by email for their postal address. In addition, a PDF copy of the questionnaire was placed on the Centre for Evidence-based Veterinary Medicine (CEVM) website, along with an explanation of the study and a printable prepaid postal return label. For organisations with multiple branches, all branches were contacted individually and the central office was also approached to obtain overall figures.

The questionnaire was pilot-tested between September and November 2010 by individuals with a variety of roles within animal welfare organisations of different sizes. Following their suggestions, the questionnaire was amended and clarified. All mailings for the actual study were sent between March and October 2011. All respondents were sent an initial copy of the questionnaire, with a covering letter and a prepaid return envelope. A pen and a chocolate were included, as incentives have been shown to increase questionnaire response rates [28]. A first reminder letter was sent to non-responders 5–12 weeks after the initial mailing. A second reminder letter was sent, with another copy of the questionnaire and a prepaid return envelope, 4–12 weeks after the first reminder. One large organisation requested that the first reminder be sent by email in its regular branch newsletter, instead of by post. All organisations that had not responded by 12 weeks after the 2^nd^ reminder were classed as non-respondents.

Respondents were asked to describe: the scope of activities of their organisation; numbers of cats and dogs cared for; the origins of these animals and outcomes of their stay; re-relinquishment rates; maximum capacity; isolation facilities; waiting lists; housing and staffing.

### Financial Information

Financial records were obtained for 2009–2010 and 2010–2011 for all organisations registered with the Charity Commission (England and Wales) [25], which fulfilled the criteria of housing or re-homing cats and/or dogs. Where their financial year did not run from January to December, records for the closest possible time period (e.g. March 2009 – February 2010) were obtained. Where organisations had several financially independent branches, all were included.

### Statistics

Descriptive statistics were compiled using Microsoft Excel (2010). As the data were non-normally distributed, medians and inter-quartile ranges (IQR) were calculated.

For those respondents who provided both a maximum capacity for dogs or cats, and a maximum capacity for their isolation facility (where present), the proportionate isolation capacity was calculated as a ratio.

$$\text{Proportionate isolation capacity=}\frac{\text{maximum isolation capacity}}{\text{maximum capacity}}$$

Similarly, the size of the waiting list, when provided, was compared to the overall maximum capacity of the organisation as a ratio.

$$\text{Proportionate waiting list=}\frac{\text{number animals on waiting list}}{\text{maximum capacity}}$$

## Results

### Response

Overall, 2,352 individuals, branches and contacts were identified as part of the sampling frame, representing 1,380 organisations (Table 1). Breed-specific rescues represented 41.8% (577/1,380) of the organisations identified.

**Table 1:** Sources of cat and dog rescue organisations identified in a search of national charitable registers, breed organisations and welfare websites

**Source**	**Number of organisations identified**
England & Wales Charity Commission (EWCC)^1^	460^*^
Office of the Scottish Charity Regulator (OSCR)^2^	35
Kennel Club	321
Governing Council of the Cat Fancy	137
Cat Chat^3^	297
Greyhound Rescue Database^4^	42
Greyhound and Lurcher Rescue^5^	42
Pawtrax^6^	30
Dog Rescue Pages^7^	5
Snowball sampling	11

^1^http://www.charity-commission.gov.uk[25]

^2^http://www.oscr.org.uk/[26]

^3^http://www.catchat.org/

^4^http://www.greyhound-data.com/bgrd/

^5^http://www.greyhoundandlurcherrescue.co.uk/

^6^http://www.pawtrax.co.uk/main/

^7^http://www.dogpages.org.uk/

Where an organisation was found in more than one location, it was attributed to the site it was first found (sources listed in the order in which they were used).

^*^The EWCC search revealed 595 animal rescues, including 135 financially independent branches of larger organisations. Therefore, overall, the EWCC contributed 460 individual organisations to the sampling frame.

Seven hundred and thirty-five unique responses were received. This included data from branches of large organisations, for which central figures were also obtained. Therefore, data from 203 branches was excluded from the analysis, and the final dataset comprised 536 respondents, a response rate of 38.8% (536/1380). Precise information was not available from one large organisation for the origin and destination of the cats in its care; therefore for these questions, data from the responding branches (100/234) were used. Not all respondents replied to every question; the number of respondents is given separately with each result. Of the 532 respondents, 243 (45.7%) were breed rescues.

Reasons for non-response were obtained from 83 respondents, and for these, the most common reasons for refusal were: a different contact for that organisation would participate (51.8%, 43/83) or that the organisation was no longer operational (42.2%, 35/83).

### Yearly total, origins and destination of cats and dogs

The total number of animals cared for in 2010 was 89,571 dogs and 156,826 cats. The median number cared for by each organisation per year was 33 for dogs (IQR 6–103), and 93 (IQR 31–215) for cats. For dogs, this figure was ‘known’ by 69% of respondents, and ‘estimated’ by 31%; for cats the figures were 59.8% and 40.2% respectively.

The most common origin of animals was relinquishment by an owner or carer, accounting for 56.3% of dogs and 45.1% of cats (Table 2). The next most common origin was as a stray, with 25.8% of dogs and 42.3% of cats presented in this way. Other sources included transfer from another welfare organisation, veterinary surgeries, and confiscation for welfare reasons. ‘Other’ categories described included animals born in the rescue, as well as those obtained from social services or police, taken in following the death of an owner, or ‘dumped’.

**Table 2:** Sources of dogs and cats presented to shelter organisations responding to a UK postal survey in 2010

	**Dogs**			**Cats**		
	**Number within organisation**		**Total (percentage)**	**Number within organisation**		**Total (percentage)**
	**Median**	**Inter-quartile range**		**Median**	**Inter-quartile range**	
Surrender by owner	20	5-66	48,770 (56.3)	56	8-117	45,899 (45.1)
Found as a stray/ lost	1	0-13	22,384 (25.8)	36	6.3-69.3	42,983 (42.3)
Another welfare organisation	0	0-1.8	10,677 (12.3)	0	0-1	1,278 (1.3)
Veterinary surgery	0	0-0	294 (0.3)	0	0-5	1,848 (1.8)
Confiscation for welfare reasons	0	0-0	3,110 (3.6)	0	0-0	8,556 (8.4)
Other	0	0-0	1,427 (1.6)	0	0-0	1,053 (1)
Total			86,662 (100)			101,617 (100)

For dogs, data were available from 301 organisations, representing 86662/89571 (96.8 %) of the dogs in the study. For cats, data were available for 197 organisations, representing 101617/156826 (64.8 %) of the cats. For one large organisation, overall central data were not available for cats; therefore data from those of its branches which responded (100/234) were used.

The most common outcome for cats and dogs was being rehomed, with 75% of dogs and 77.1% of cats finding new owners (Table 3). The second most common outcome was euthanasia, with 10.4% of dogs and 13.2% of cats being humanely destroyed. For cats, 3.2% were released, in conjunction with trap-neuter-return (TNR) projects. A relatively small proportion of dogs and cats (7.1% and 1.4% respectively) were reunited with their owners, with the remainder having died, been transferred to another organisation, or remaining in the care of the organisation at the time of response.

**Table 3:** Destinations of dogs and cats presented to shelter organisations responding to a UK postal survey in 2010

	**Dogs**			**Cats**		
	**Number within organisation**		**Total (percentage)**	**Number within organisation**		**Total (percentage)**
	**Median**	**Inter-quartile range**		**Median**	**Inter-quartile range**	
Reunited with owner	0	0-0	6,222 (7.1)	1.8	0-4.4	1,331 (1.4)
Rehomed	31	8-100	65,519 (75)	84.5	18.6-179.1	75,860 (77.1)
Still in organisation’s care	0	0-4	5,638 (6.5)	2	0-14.3	3,284 (3.3)
Sent to another welfare organisation	0	0-0	625 (0.7)	0	0-0	609 (0.6)
Euthanased	0	0-2	9,059 (10.4)	1	0-3.5	12,989 (13.2)
Released (e.g. ferals)	not applicable			0	0-2	3,512 (3.6)
Died	0	0-0	154 (0.2)	0	0-1	561 (0.6)
Other	0	0-0	131 (0.1)	0	0-0	202 (0.2)
Total			87,348 (100)			98,348 (100)

For dogs, data were available from 305 organisations, representing 87348/89571 (97.5 %) of the dogs in the study. For cats, data were available from 194 organisations, representing 98348/156826 (62.7 %) of the cats in the study. For one large organisation, overall central data were not available for cats; therefore data from those of its branches which responded (100/234) were used. Trap-neuter-return is not practised for dogs in the UK.

### Re-relinquishment

Respondents were asked what percentage of animals was later returned (re-relinquished) to them. For dogs, 303 respondents replied, with a median re-relinquishment percentage of 1% (IQR 0–5). For cats, 190 respondents replied, with a median re-relinquishment percentage of 1% (IQR 0–3).

### Capacity

**Figure 1:**
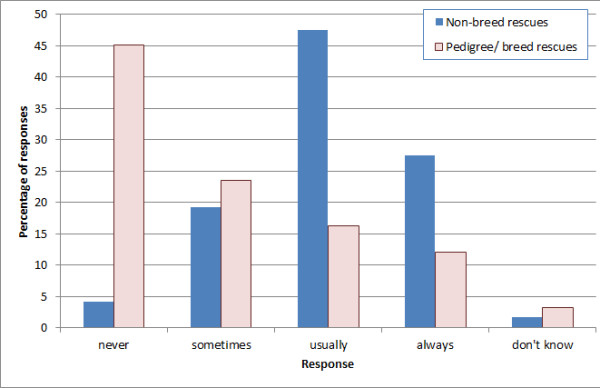
**Frequency of being full to capacity (dogs). **Dataset n = 536; respondents n = 311 of which pedigree/ breed rescues n = 191; all others n = 120.

**Figure 2:**
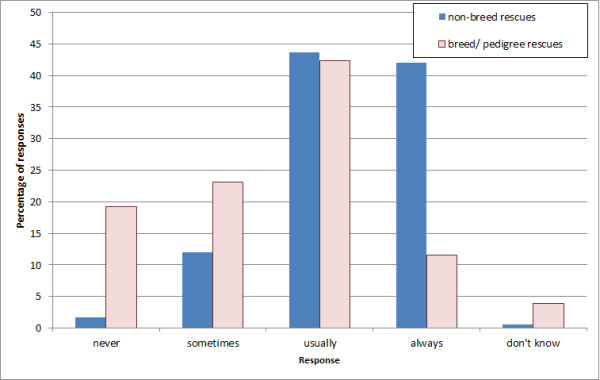
**Frequency of being full to capacity (cats). **Dataset n = 536; respondents n = 209 of which pedigree/ breed rescues n = 26; all others n = 183.

Of the 536 respondents, 259 (48.3%) had dogs in their care at the time of survey. The total number of dogs housed at the time of reply was 10,630, with the median number housed being 10 (IQR 4–26). Similarly, 208 (38.8%) organisations had cats in their care at the time of reply, housing a total of 18,053, with a median number housed of 27 cats (IQR 11–45). The median maximum capacity for dogs was 10 (IQR 4–25), and for cats was 30 (IQR 14–55). Respondents were asked how often they were full to capacity (Figure 1 and Figure 2). Breed rescues differed substantially from other rescues, as they were frequently not full.

### Proportionate maximum isolation capacity

Most of the responding organisations had an isolation facility, with 73.8% (239/324) stating one was present for dogs, and 83% (181/218) for cats. For both dogs and cats, the median proportionate isolation capacity was 0.1, signifying that 10% of the housing capacity was available for isolation.

### Waiting list

Of 343 respondents, 152 (44.3%) had a waiting list for dogs. One hundred and forty-seven respondents provided the number of dogs on their waiting list, which overall totalled 3,410 dogs. One hundred and thirty-five organisations provided both waiting lists and maximum capacity figures for dogs. The median proportionate waiting list was one third of the size of the maximum capacity, and the largest was 16.7 times the maximum capacity (i.e. there were 16.7 times the number dogs on the waiting list as the organisation could actually house at any one time).

For cats, 134/246 (62%) respondents held a waiting list, and numbers were provided by 127 respondents, totalling 4,338 cats. In the 114 organisations for which both maximum capacity and waiting list size were stated, the median proportionate waiting list was half the size of the maximum capacity, with the largest list being 6.7 times the maximum capacity of the organisation.

### Housing

Data on the locations of the animals in care at the time of the survey were available for 10,548 dogs and 13,487 cats. Both dogs and cats were most likely to be housed in kennels or catteries owned by the organisation with 10% (1,054/1,548) of the dogs housed in this way as compared to 47.2% (6,373/13,487) of the cats. Of the remaining animals, most were housed in private kennels or catteries.

### Scope of organisation

The most common services provided were housing (52.5% of organisations handling dogs, 52.9% of organisations handling cats), rehoming (62.1% dogs, 54.2% cats) and financing veterinary care (46.9% dogs, 41.9% cats). Less common activities included trap-neuter-return and providing financial assistance to other organisations. Other activities mentioned included providing pet care, rehoming and behavioural advice, social and community activities and education.

### Staffing

When asked about staffing levels, 445 respondents replied. A total of 19,302 staff members were employed by these respondents, of whom 2,758 (14.3%) were paid full-time, 1,125 (5.8%) paid part-time, 834 (4.3%) were voluntary full-time and 14,585 (75.6%) voluntary part-time.

### Financial resources

Financial records were available in 2009 for 532/595 (89.4%) and in 2010 for 519/595 (87.2%) of the organisations registered with the England and Wales Charities Commission. These records included 460 separate organisations, and an additional 135 financially independent branches, as denoted by an individual registration number and separate accounts. Collectively, the total expenditure of the organisations registered with the EWCC in 2009–2010 was £328,241,667 (median £48,746, IQR £17,684-£147,873), and in 2010–11 was £339,937,756 (median £49,987, IQR £19,758-£150,282).

## Discussion

This study provides an insight into the UK’s un-owned cat and dog population in 2010, and its dynamic relationship with the owned population. The study has shown that at least 89,571 dogs and 156,826 cats were cared for in 2010 by the 536 organisations surveyed. These numbers are of a similar scale to those identified by Clark et al. in 2009 [4], and provide a valuable comparison, both in terms of absolute numbers, and contrasting techniques used. The present study also adds substantially by including data on logistics, encompassing housing use, isolation facilities, finances and staffing, as well as re-relinquishment data and waiting lists.

In the present study, over 9,000 dogs were reported as having been euthanased. However it is likely that this represents under-reporting of the true figure. The Dogs Trust’s annual stray dogs survey [3] identified the numbers of stray dogs handled by local authorities in the UK between April 2010 and March 2011. Of an estimated total of 126,176 stray dogs, 7121 (6%) were euthanased by local authorities. If these are added to the numbers of dogs identified in the present study, this suggests that in excess of 16,000 dogs may be euthanased per annum as strays or shelter animals in the UK. These figures may however partially overlap with those in the present study, in that those organisations reporting the origin of dogs as ‘stray’ may include those taken from local authority kennels. This may also explain the relatively low numbers of dogs in the current study which were reunited with their owners. Many shelters take in dogs following their statutory 7-day holding period in council kennels. Therefore some dogs may have been reclaimed directly from council kennels during this period. No such statutory minimum holding period applies to cats.

In this study, almost 13,000 cats were reported as being euthanased. Exploration of reasons why these animals were destroyed fell outside the aims of the present study. It is probable that a proportion were euthanased because they were suffering from intractable illness or severe behavioural problems. It is, however, also possible that, given the scale of the un-owned population, some healthy, potentially re-homeable animals had to be destroyed due to high demand for spaces. The numbers of animals euthanased in 2010 were considerably higher than the numbers identified in 2009 by Clark et al. [4]. It is possible this represents a genuine increase in euthanasia. Alternatively, the two studies may have captured different respondents, with the present study including some organisations with a much higher euthanasia rate. Further research is warranted into how decisions are made regarding euthanasia of un-owned animals.

Approximately half of the cats and dogs had been relinquished by their owner or carer. Although the reasons for relinquishment were not explored in the present study, previous studies have shown pet relinquishment to be multifactorial in origin. It has been suggested that animals obtained at low or no cost are at increased risk of relinquishment, presumably because they may represent a less committed purchase [12]. Behavioural problems have been shown to increase the risk of relinquishment [29], and relinquishing owners have been shown to be relatively poorly informed on basic behaviour. For example, in one study >50% of relinquishing cat and dog owners thought that their animals misbehaved ‘out of spite’ [30]. The financial climate may also play a part. Although one study in a Chicago shelter found that the recent economic downturn only slightly affected relinquishments [24], numerous UK [David Yates (RSPCA; pers. comm.), Maggie Roberts (Cats Protection; pers. comm.) and Mandy Jones (Blue Cross; pers. comm.)] and US [31] welfare and shelter organisations have expressed concern that greater demands are being made of them as a result of the current economic climate.

A relatively small proportion of the animals entered shelters from sources other than straying and relinquishment. It is evident that some organisations transfer animals between themselves, although the small numbers involved suggest that this is not common. Another origin was confiscation for welfare reasons, with over 11,000 dogs and cats admitted in this way. However, this aspect of welfare work was carried out by a relatively limited number of participants (as shown by an interquartile range of 0–0), further highlighting the variation in the services provided by participants.

This diversity between organisations has been suggested in previous research [4], and can also be inferred by the variation seen in re-relinquishment rates in the survey respondents. This may reflect philosophical divergences between organisations - some enforce a contract that states a re-homed animal must always be returned to the shelter if no longer wanted, whereas others do not. Reasons for re-relinquishment were not explored in the present study. However it has been previously shown that behavioural problems are a significant risk factor for both initial and re-relinquishment [32,33]. Disease in the immediate post-adoption period has also been correlated with re-relinquishment [34].

The presence of an isolation and quarantine facility is important in the control of infectious diseases in shelters [35-37]. Most respondents had some form of isolation facility; however 26.2% and 17% had none, for dogs and cats respectively. In addition to the implications for those in the shelter environment, animals incubating shelter-acquired infectious disease and developing clinical signs subsequent to rehoming have been shown to be at increased risk of re-relinquishment [38].

Most of the cat rescues, and most of the non-breed dog rescues, were ‘usually’ or ‘always’ full to capacity. Many organisations held a waiting list, which was often much larger than their actual capacity. A degree of response bias is possible; organisations with a longer list may be more inclined to reply. Regardless of this, these figures do demonstrate the overwhelming pressures under which some organisations operate.

The resources used in the care of the animals described by this study are substantial. The organisations surveyed employed a total of 19,302 staff, of which 79.9% worked on a full or part time voluntary basis. In addition, a total of £340 million was spent on the care of un-owned animals by the 519 charities whose records were available from the EWCC. Unregistered charities have no legal requirement to publish their accounts but clearly the expenditure of the organisations outwith the EWCC would be in addition to this total sum. Collectively then, a large number of man-hours and a substantial financial sum are expended on un-owned animals in the UK.

An important limitation of this study lies in the fact that it was not possible to gather data from every organisation engaged in the care of un-owned cats and dogs. There is currently no statutory licensing requirement for any ‘shelter’ organisation in the UK and therefore no central register. Despite extensive searches to construct the sampling frame, it is likely that there are organisations which were not identified by this study. The overall response rate of 38.8% included overall statistics for the high-profile large organisations. It is likely that many of the non-responding organisations are smaller; however, it is difficult to extrapolate the data further without more information about them. Even the responding organisations often did not keep complete records, so some of the data supplied were estimated. It is also likely that there are un-owned animals which will not be captured in this study. For example, a report in 1998 stated that 140,000 pets were given up by older people entering residential care [39], suggesting that there may be other animals becoming un-owned which have not been captured by the present data. Therefore the figures presented within this study represent under-reporting of the true numbers of un-owned cats and dogs in the UK.

## Conclusions

This study attempted to characterise an under-studied and hard to reach population of animals. We have shown that the un-owned dog and cat population in the UK is both extensive and diverse. Despite substantial quantities of manpower and money expended on these animals, it appears that at this time there is still a continual flow of animals out of ownership and into the guardianship of rescues and shelters. It is clear that further understanding of the reasons for this flow and how targeted interventions may affect the size and character of the un-owned population is vital if we are to prevent this continued cycle of over-production and relinquishment.

## Competing interests

Financial competing interests

In the past five years have you received reimbursements, fees, funding, or salary from an organization that may in any way gain or lose financially from the publication of this manuscript, either now or in the future? Is such an organization financing this manuscript (including the article-processing charge)? If so, please specify.

No

Do you hold any stocks or shares in an organization that may in any way gain or lose financially from the publication of this manuscript, either now or in the future? If so, please specify.

No

Do you hold or are you currently applying for any patents relating to the content of the manuscript? Have you received reimbursements, fees, funding, or salary from an organization that holds or has applied for patents relating to the content of the manuscript? If so, please specify.

No

Do you have any other financial competing interests? If so, please specify.

No

Non-financial competing interests

Are there any non-financial competing interests (political, personal, religious, ideological, academic, intellectual, commercial or any other) to declare in relation to this manuscript? If so, please specify.

No

## Authors’ contributions

JS and RD conceived of the study. JS carried out the survey, performed the data analysis and drafted the manuscript. RD, MLB and MD participated in the design of the questionnaire, data analysis and preparation of the final manuscript. All authors read and approved the final manuscript.
